# Formulation and Evaluation of Liposome-Encapsulated Phenolic Compounds from Olive Mill Waste: Insights into Encapsulation Efficiency, Antioxidant, and Cytotoxic Activities

**DOI:** 10.3390/molecules30112351

**Published:** 2025-05-28

**Authors:** David Camilleri, Karen Attard, Frederick Lia

**Affiliations:** Applied Research & Innovation Centre, Malta College of Arts, Science & Technology MCAST, 9032 Paola, PLA, Malta; david.camilleri.c11052@mcast.edu.mt (D.C.); karen.attard@mcast.edu.mt (K.A.)

**Keywords:** encapsulation efficiency, phenolic compounds, cytotoxicity, olive mill waste

## Abstract

Phenolic extracts obtained from the solid by-products of olive oil production (collectively referred to as “olive mill waste”) were encapsulated in phosphatidylcholine/cholesterol liposomes using the thin-film hydration method. This study examines how lipid composition, cholesterol content, and two different approaches to introducing phenolics affect the efficiency with which these bioactive compounds are encapsulated. ‘Bidni’ and ‘Bajda’ cultivars are two main olive cultivars found in Malta. ‘Bajda’ is an example of a variety exhibiting leucocarpa. Unlike typical olives, leucocarpa drupes remain white during ripening due to silenced anthocyanin-producing genes. These two extracts were tested for encapsulation efficiency and then evaluated for in vitro cytotoxicity against human leukemia cells. Our results show that increasing the amount of cholesterol in the liposomes generally improved the retention of phenolic compounds, whereas the encapsulation route (i.e., inclusion with the lipids versus hydration medium) had differential effects on specific phenolics. Additionally, liposomal encapsulation provided more potent cytotoxic activity over 48 h compared to the free extract, suggesting that liposomes can enhance and prolong the delivery of bioactive compounds from this agri-food waste.

## 1. Introduction

Olive (*Olea europaea* L.) oil production is culturally tied to the Mediterranean region and is a signature component of most Mediterranean diets. As a result, olive cultivation and production of its oil also has strong historical links to the Maltese Islands and ancient olive cultivars, primarily ‘Bidni’, ‘Bajda’ and ‘Malti’, are still alive to the present day [1].

The ‘Bajda’ and ‘Bidni’ are monocultivars, while ‘Malti’ consists of ancient, geographically isolated varieties [2]. ‘Bajda’ exhibits leucocarpa, where the drupe remains white during ripening due to silenced anthocyanin-producing genes [3].

Olive mill waste is a growing environmental issue in Mediterranean countries due to their high production volume and phytotoxicity. Despite this, their high proportion of organic matter, which is mainly composed of phenolics, lipids and organic acids, means that a wide range of valuable compounds can be recycled and re-purposed. Several authors have already explored revalorization of olive mill waste such as second oil extraction, combustion, gasification, anaerobic digestion, composting and solid fermentation [4]. Their high content of polyphenols, which are a group of organic compounds renowned for antioxidant activity, makes olive mill wastes of particular interest to researchers who seek to exploit them for their health and therapeutic benefits. In a collective effort to investigate the revalorization of Maltese OMWs, a sister study to this research project [5] revealed a significant presence of ligstroside, p-coumaric acid and apigenin-7-glucoside in addition to the signature compounds 3-hydroxytyrosol, oleuropein, oleacein and tyrosol. Aside from their well-known status as potent antioxidants, these compounds have demonstrated several other potential uses in the field of therapeutics such as anti-cancer [6,7], antimicrobial [8], anti-inflammatory [9,10], cardioprotective [11] and neuroprotective [12,13].

Due to their structural versatility and their ability to encapsulate and transport both hydrophilic and hydrophobic compounds, liposomes have been gaining notable attention as researchers seek to exploit their physiochemical characteristics into novel transport mechanisms for delivering biologically active compounds. This concept has also gained some application in cosmetic, pharmaceutical, food and agricultural industries for delivery of unstable compounds such as antioxidants and antimicrobials [14]. Liposomal encapsulation delivery also boasts several advantages such as biocompatibility, low toxicity, targeted delivery and protection of the encapsulated compound from harsh environmental conditions. Most notably, liposomes have shown some success as an alternative drug delivery system for a large spectrum of treatments including cancer, antibiotic, antifungal and vaccination [15]. In principle, the technique of liposomal encapsulation can be applied to the compounds of interest found in olive mill waste, with the scope of overcoming their primary disadvantages, which include poor solubility, chemical instability and the difficulty to reach their intended biological target [16]. Despite other studies [17,18,19] having already initiated investigations related to phenolic compound encapsulation, specific research regarding the encapsulation of olive mill waste phenolics and their applications is still in its infancy, with few papers being published [20,21,22]. Given the importance of sustainable valorization strategies for olive mill wastes (OMWs), this study aims to investigate how different liposomal formulations and preparation methods affect the encapsulation efficiency of crude OMW phenolics. In doing so, we compare both non-liposomal and liposomal preparations derived from two Maltese cultivars (‘Bidni’ and ‘Bajda’), examining whether variations in cultivar or in liposome composition influence encapsulation efficiency and in vitro activity. We also evaluate the cytotoxic effects of these encapsulated extracts on the HL-60 human promyelocytic leukemia cell line. Overall, the work is divided into two main components: (i) sampling, extraction, and preparation of both free and liposome-encapsulated OMW phenolics, and (ii) characterization of their encapsulation efficiency and bioactivity (via MTT assays). Through this approach, we provide insights into the potential of liposomal encapsulation to enhance the antioxidant and anticancer properties of OMW phenolics.

## 2. Results

### 2.1. Comparative Analysis of the Two Methods of Encapsulation

To evaluate the influence of the three independent factors—Cultivar (‘Bidni’ vs. ‘Bajda’), Formulation Ratio (80:20 vs. 70:30), and Processing Method (Method A vs. Method B)—ANOVA was conducted. This approach enables the assessment of the main effects. These test whether each factor independently affects the response variable(s). The analysis focused on identifying the main effects of three independent factors: Cultivar (‘Bidni’ vs. ‘Bajda’), Formulation ratio (80:20 vs. 70:30), and Processing Method (Method A vs. Method B). The effect of cultivar was assessed to determine variations in response between ‘Bidni’ and ‘Bajda’, independent of formulation and method. The formulation effect evaluated whether the 80:20 ratio differed significantly from the 70:30 ratio across all treatments. The method effect examined differences in outcomes between Method A and Method B, irrespective of cultivar and formulation.

**Figure 1:**
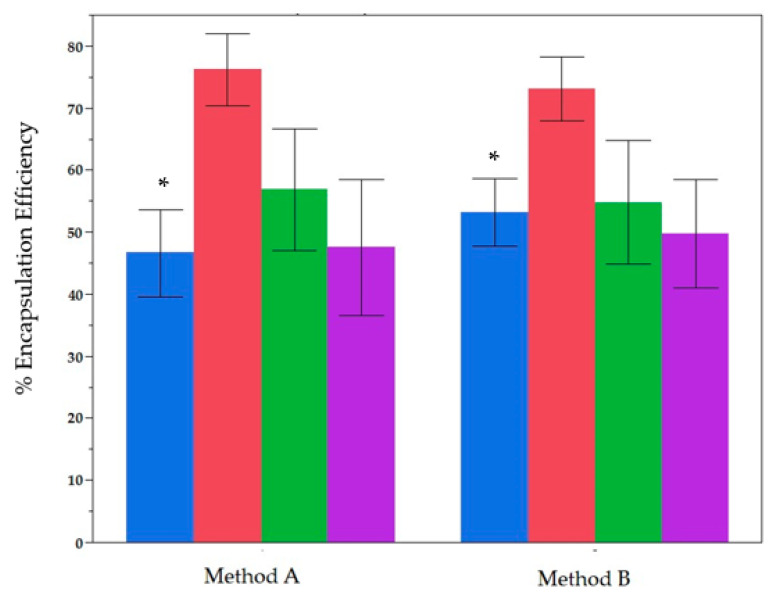
Explanation of encapsulation efficiency (EE%) and antioxidant activity for two liposome preparation methods. Blue: TPC, Red: TFC, Green: TdOPC, Purple: CUPRAC. Bars show mean ± standard deviation of 24 samples for Method A and Method B. (*) indicate significant difference *p* < 0.05 Post-Hoc (Mann Whitney U Test).

This section compares Method A—where phenolic extracts are co-dissolved in the lipid–chloroform mixture prior to solvent removal—with Method B—where extracts are introduced during the hydration phase in phosphate-buffered saline (PBS). As illustrated in Figure 1, encapsulation efficiency (EE%) was evaluated for total phenolic content (TPC), total flavonoid content (TFC), total ortho-diphenolic content (TdOPC), and CUPRAC across both cultivars (‘Bidni’ and ‘Bajda’) and the two phosphatidylcholine–cholesterol formulations (Formulation 1 vs. Formulation 2).

According to the Kruskal–Wallis test, TPC is significantly higher when Method B is used (*p* < 0.05). In contrast, TFC, TdOPC, and CUPRAC show no statistically significant differences (*p* > 0.05) between the two methods, although there are minor trends (e.g., TFC is marginally higher under Method A). Overall, these findings suggest that Method B significantly improves the encapsulation of total phenolic content but does not offer a statistically verifiable advantage for flavonoids or antioxidant indices compared to Method A.

### 2.2. Comparative Analysis of the Two Formulations Used for Encapsulation

**Figure 2:**
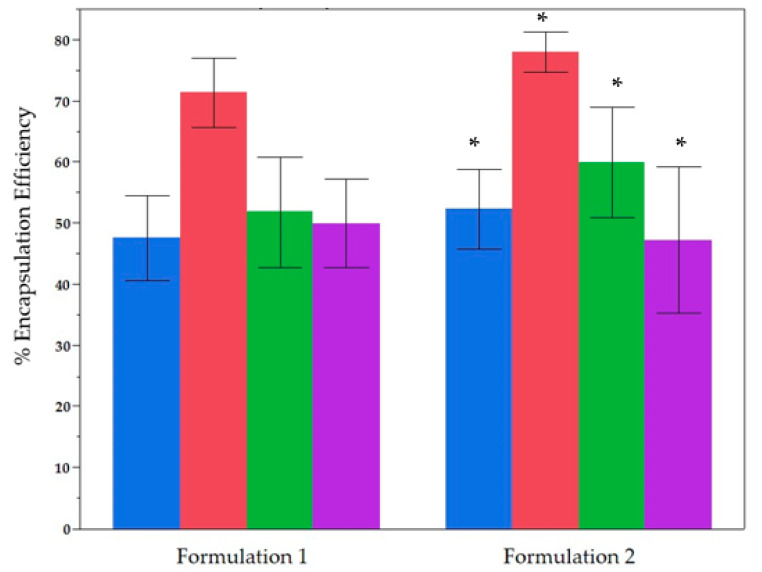
Explanation of the encapsulation efficiency (EE%) and antioxidant activity for two liposomal formulations Formulation 1 (80:20) and Formulation 2 (70:30). Blue: TPC, Red: TFC, Green: TdOPC, Purple: CUPRAC. Bars show mean ± standard deviation of 24 samples for Formulation 1 and Formulation 2. (*) indicate significant difference *p* < 0.05 Post-Hoc (Mann Whitney U Test).

Two distinct phosphatidylcholine-to-cholesterol (PC:Chol) ratios were evaluated to determine the effect of varying cholesterol content on encapsulation efficiency. Formulation 1 (80:20) was selected to examine the impact of reduced cholesterol, whereas Formulation 2 (70:30) reflects a commonly reported 2:1 ratio. Figure 2 compares the encapsulation efficiencies (EE%) of (TPC, TFC, TdOPC, and CUPRAC across both formulations, encompassing Method A and Method B as well as the Bidni and Bajda cultivars. According to the Kruskal–Wallis test, Formulation 2 achieves significantly higher EE% for TPC (*p* = 0.035), TFC (*p* = 0.001), and TdOPC (*p* = 0.014) compared with Formulation 1. By contrast, CUPRAC exhibits no significant difference (*p* > 0.05) between these two formulations. Overall, these findings underscore the enhanced encapsulation performance of Formulation 2—particularly when paired with Method B—in delivering phenolic compounds from both cultivars.

### 2.3. Comparative Analysis of Encapulsation Effiencies for Two Different Cultivars

The Maltese olive cultivars ‘Bidni’ and ‘Bajda’ exhibit potentially distinctive phenolic profiles, with ‘Bidni’ typically noted for its robust flavor and comparatively higher oleuropein content, and ‘Bajda’ reportedly containing a broader range of flavonoids and secondary metabolites. Given these unique phytochemical characteristics, both cultivars were investigated to determine whether inherent variations in their phenolic composition would influence encapsulation outcomes.

**Figure 3:**
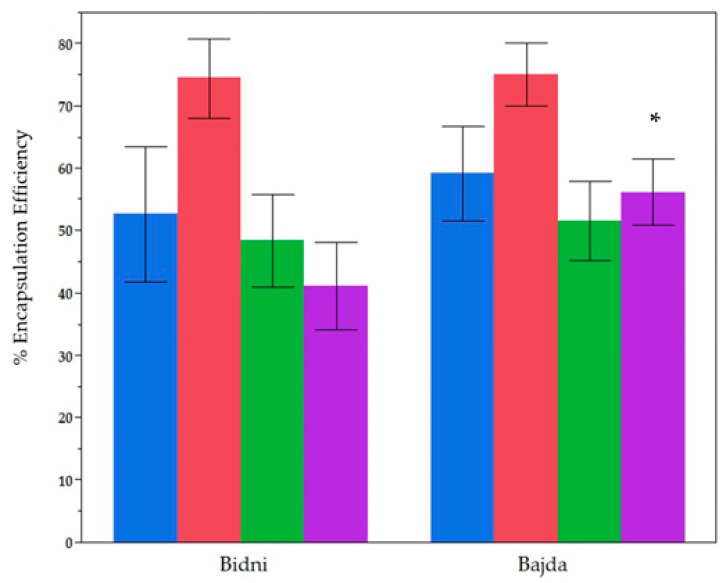
Explanation of encapsulation efficiency (EE%) and antioxidant activity of liposomal formulations derived from different cultivars. Blue: TPC, Red: TFC, Green: TdOPC, Purple: CUPRAC. Bars show mean ± standard deviation of 24 samples for Bidni and Bajda. (*) indicate significant difference *p* < 0.05 Post-Hoc (Mann Whitney U Test).

Figure 3 illustrates the encapsulation efficiencies (EE%) for total phenolic content (TPC), total flavonoid content (TFC), total ortho-diphenolic content (TdOPC), and CUPRAC across ‘Bidni’ and ‘Bajda’, using Methods A/B (encapsulation approaches) and Formulations 1/2 (cholesterol ratios). A Mann–Whitney U test confirms that TPC (*p* = 0.171), TFC (*p* = 0.8381), and TdOPC (*p* = 0.0801) do not differ significantly between the cultivars. However, CUPRAC is significantly higher (*p* = 0.0001) for ‘Bajda,’ suggesting an enhanced antioxidant capacity in comparison to ‘Bidni’, while the encapsulation of other phenolic indices remains broadly similar. This result highlights the likelihood that cultivar-specific composition may selectively influence certain antioxidant metrics.

### 2.4. Cytotoxicity (IC_50_) After 48 H with Respect to Method, Formulation and Cultivar

**Figure 4:**
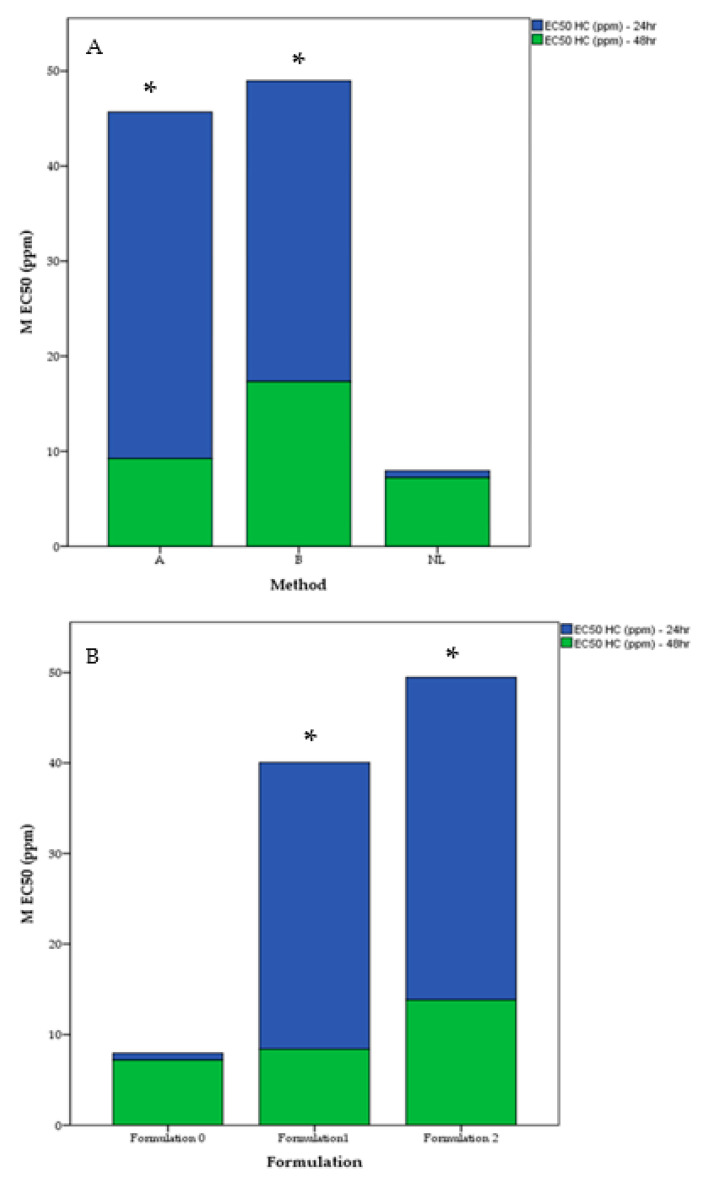
(**A**) Effect of Formulation (0, 1, or 2) on IC_50_ (ppm) at 24 h (blue) and 48 h (green) in HL-60 cell assays. Formulation 0 is non-liposomal, while Formulations 1 and 2 differ in phosphatidylcholine:cholesterol ratios (80:20 vs. 70:30). (*) indicates significant difference *p* < 0.05 Post-Hoc (Mann Whitney U Test). (**B**): Effect of Encapsulation Method (A, B, NL) on the EC_50_ (ppm) at 24 h (blue portion) and 48 h (green portion) in HL-60 cell assays. Method A co-dissolves the extract in the lipid-chloroform mixture, Method B adds the extract during hydration, and NL is non-liposomal. (*) indicate significant difference *p* < 0.05 Post-Hoc (Mann Whitney U Test).

Figure 4A shows that at 24 h, a significant difference in IC₅₀ values was observed among the formulations (*p* < 0.05), while by 48 h, no significant differences remained (*p* > 0.05). Post-hoc analysis showed that Formulation 0 (non-liposomal) differed significantly from Formulation 2 (*p* < 0.05) and marginally from Formulation 1, whereas Formulations 1 and 2 did not significantly differ. At 24 h, Formulation 0 exhibited the lowest IC₅₀, indicating faster cytotoxicity, whereas Formulation 2 (70:30 PC:Chol) had the highest IC₅₀, suggesting a delayed release due to its higher cholesterol content. Formulation 1 (80:20 PC:Chol) displayed an intermediate IC_50_, often not significantly different from the other formulations. By 48 h, all formulations showed similar IC₅₀ values, indicating that early differences in membrane composition and release kinetics dissipate with prolonged exposure. The sustained release observed in Formulation 2 may be beneficial for applications requiring prolonged cytotoxic effects.

Figure 4B revealed a significant difference in IC₅₀ values among the encapsulation methods at 24 h (*p* = 0.021), while no significant differences were observed at 48 h (*p* = 0.124). Pairwise comparisons showed that the non-liposomal control (NL) differed significantly from both Method B (*p* = 0.045) and Method A (*p* = 0.026), indicating that NL achieved faster cytotoxicity at lower concentrations. However, no significant difference was found between Methods A and B (*p* = 1.000), suggesting similar early stage IC₅₀ values for these liposomal approaches. By 48 h, IC_50_ values for all methods implies the initial differences in cytotoxic response diminished with prolonged incubation. These findings confirm that, at 24 h, non-liposomal extracts require lower concentrations to reach 50% inhibition than either liposomal method. However, Method A and Method B do not differ significantly from each other in the short term, and all methods produce comparable results by 48 h.

**Figure 5:**
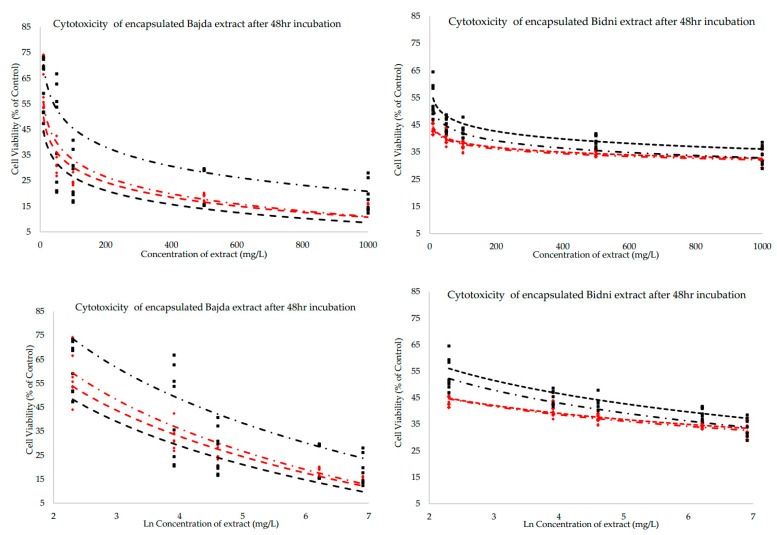
Cytotoxicity of encapsulated Bajda (**Left**) and Bidni (**Right**) extracts after 48 h of incubation, plotted as cell viability (% of control) versus extract concentration (mg/L) (**Top**) and Ln of concentration (**Bottom**). Different formulations are represented by colors, and the method of preparation is indicated by the line pattern. **— —** Method A Formulation 1; **— · —** Method A Formulation 2; **— —** Method B Formulation 1; **— · —** Method B Formulation 2.

The data presented in Figure 5 illustrate the cytotoxic effects of encapsulated extracts derived from the Bidni and Bajda cultivars following a 48-h incubation period. Both sets of figures depict the correlation between extract concentration (mg/L) and cell viability (% of control), revealing a dose-dependent decline in cell viability across all tested methods and formulations. In the extracts from both Bidni and Bajda, cell viability diminishes logarithmically as extract concentration increases, suggesting that higher concentrations yield more pronounced cytotoxic effects. Method A consistently demonstrates enhanced cytotoxicity compared to Method B, as indicated by lower cell viability at equivalent concentrations. This pattern is consistent across both cultivars. Furthermore, Formulation 2 exhibits more potent cytotoxic effects than Formulation 1 in both methodologies, with the most significant decrease in cell viability occurring in Method B utilizing Formulation 2. Although both cultivars display comparable dose-response relationships, the Bajda extracts exhibit marginally greater cytotoxicity than the Bidni extracts, particularly evident in Method B with Formulation 1.

## 3. Discussion

The presence of cholesterol in liposomal formulations significantly impacted encapsulation efficiency, with Formulation 2 (containing 10% more cholesterol) consistently outperforming Formulation 1 in TPC, TFC, and TdOPC assays. Encapsulation efficiencies improved by 5%, 7%, and 8% for TPC, TFC, and TdOPC, respectively, in Formulation 2. Cholesterol’s role in enhancing liposomal integrity and colloidal stability [23], along with its contested impact on membrane fluidity [24], likely reduces susceptibility to mechanical stress during downsizing. Cholesterol also impedes hydrophilic molecule passage through the bilayer [25], limiting leakage of polar phenolic compounds, as supported by another author [26].

Interestingly, in the CUPRAC assay, neither formulation showed statistically significant differences in encapsulation efficiency, although mean EE% favored Formulation 1. The CUPRAC assay’s inability to differentiate between phenolic and non-phenolic reducing compounds, such as Fe(II) ions [27], suggests that cholesterol’s influence is compound-specific. Cholesterol’s role in suppressing inorganic ion permeation [24] might enhance retention of such compounds but hinder their initial encapsulation, as seen with Formulation 2. This could result from electrostatic repulsion and reduced passive diffusion of polar inorganic ions. Overall, the findings align with previous studies [26,28,29], demonstrating that higher cholesterol content generally enhances encapsulation efficiency but with exceptions depending on compound characteristics.

The results show that Method A is more effective for flavonoid encapsulation, whereas Method B demonstrates greater efficiency for TPC, a fraction largely composed of hydrophilic compounds. This difference aligns with findings by [30], who note that drug loading into liposomes depends on compound polarity: lipophilic compounds are typically co-dissolved in organic solvents before evaporation, while hydrophilic compounds are introduced through the hydration medium. Supporting this, another author [31] reported that hydrating highly polar compounds yields significantly higher encapsulation efficiencies at a large scale compared to incorporating them into the dry film before hydration.

Method A is hypothesized to be more favorable for encapsulating lipophilic phenolic compounds due to its mechanism of water removal and subsequent hydration. During water removal, non-polar phenolics align with the lipophilic alkyl chains in the dried lipid film via strong hydrophobic interactions, embedding these compounds within the bilayer. Conversely, hydrophilic phenolics interact with the polar heads of the phospholipids or aggregate outside the bilayer. Upon hydration, non-polar phenolics, already integrated into the bilayer, remain stabilized, while hydrophilic phenolics dissolve into the aqueous medium unless entrapped during bilayer collapse.

**Figure 6:**
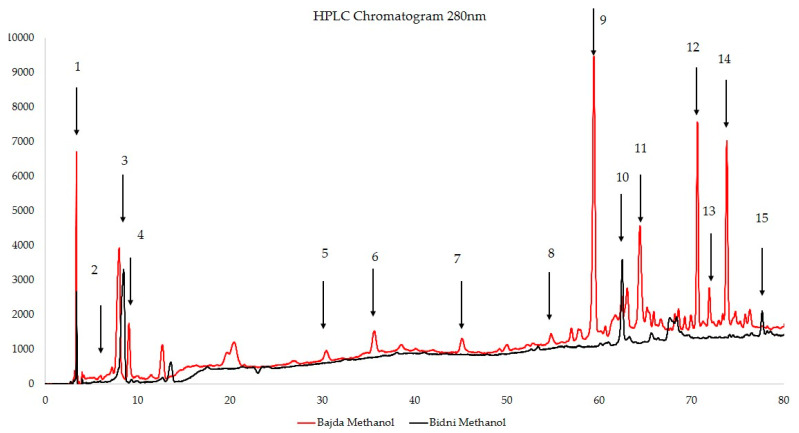
Representative HPLC chromatograms (280 nm) adopted from [5] of methanolic extracts from ‘Bajda’ (red) and ‘Bidni’ (black) olive cultivars. Peaks 1–15 are labeled according to the major phenolic compounds identified via comparison with authentic standards (see Table 1). Retention times and relative peak intensities illustrate cultivar-specific differences in phenolic composition.

The thermodynamic challenge of hydrating non-polar solids requires vigorous agitation during hydration to emulsify the suspension. This step overcomes immiscibility and facilitates the formation of spherical liposomes. The agitation also promotes the collapse of phospholipid bilayers, entrapping some solvated polar phenolic compounds while stabilizing non-polar compounds pre-embedded in the bilayer. This dual mechanism explains Method A’s effectiveness in encapsulating phenolic compounds with higher lipophilicity. Method B appears less suitable for encapsulating lipophilic phenolic compounds but better suited for hydrophilic fractions due to its reliance on co-introducing compounds with the hydration medium. In this mechanism, polar phenolics distribute more evenly along the hydrophilic phospholipid heads between bilayer sheets. However, non-polar phenolics, being less soluble in aqueous media, tend to aggregate, especially when stable in their unionized form. Unlike Method A, these compounds lack pre-integration into the bilayer, making it more difficult for them to embed during hydration. Additionally, polar forces from the phospholipid heads may repel these non-polar aggregates, further reducing their encapsulation. Polar phenolic compounds, on the other hand, can self-encapsulate via passive diffusion into the bilayer. This process is influenced by cholesterol content, which modulates membrane fluidity. In Method B, vigorous mixing during emulsification helps overcome electrostatic repulsions and stabilizes the system. During bilayer collapse, some solvated polar phenolics are entrapped, though the primary mechanism for non-polar phenolic encapsulation relies on the energy input from agitation. Representative HPLC chromatograms (280 nm) adopted from [5] of methanolic extracts from ‘Bajda’ and ‘Bidni’ olive cultivars presented in Figure 6 and Table 1 identified the abundant presence of phenolic compounds such as oleacein, oleuropein, apigenin 7-glucoside, and 3-hydroxytyrosol in both the ‘Bidni’ and ‘Bajda’ extracts. Additional phenolics, including quercetin, tetrahydroxyflavone, and vanillin, were more prominent in the ‘Bajda’ cultivar, while 2-(4-hydroxyphenyl) ethanol and p-coumaric acid were abundant in both cultivars.

Figure 6 reveals distinct hydrophilicity and lipophilicity profiles for these compounds, as summarized in Table 2. The TPSA and XLogP values provide insight into the encapsulation behavior of these phenolic compounds, with lipophilic compounds favoring lipid bilayer integration and hydrophilic compounds relying on hydration-based mechanisms. These distinctions are crucial for optimizing liposomal formulations tailored to the compound’s physicochemical properties. Hydrophilic compounds, such as oleuropein and apigenin 7-glucoside, are likely better encapsulated through the hydration medium (Method B), favoring their interaction with the polar heads of the bilayer. Lipophilic compounds, such as tetrahydroxyflavone and quercetin, align more favorably with lipid alkyl chains during film formation, aligning with Method A’s mechanism.

3-Hydroxytyrosol and oleuropein are predominantly hydrophilic, while oleacein exhibits greater lipid affinity, as indicated by their TPSA and XLogP values. Apigenin 7-glucoside shows amphiphilic properties, explaining its unique behavior during encapsulation. These distinctions provide insight into the superior performance of Method B for total phenolic compounds (TPC), as its mechanism aligns better with the encapsulation of hydrophilic phenolics, which dominate the matrix. Method B’s higher encapsulation efficiency for TPC underscores its suitability for polar phenolic compounds.

Conversely, Method A demonstrated superior encapsulation efficiency for total flavonoids and ortho-diphenols (TdOPC) over Method B, respectively. Although the difference in TdOPC efficiency was not statistically significant, Method A’s effectiveness was particularly evident in the ‘Bajda’ Formulation 1 sample, where it achieved a 10% increase in flavonoid encapsulation efficiency and a 7% increase for ortho-diphenols. This result is likely influenced by the presence of oleacein, a lipophilic orthodiphenolic secoiridoid, which contributed to the enhanced encapsulation efficiency of lipophilic compounds in Method A.

Phenolics like 2-(4-hydroxyphenyl) ethanol and p-coumaric acid, with hydrophilic heads and lipophilic midsections, may integrate into the lipid bilayer, akin to cholesterol. Their incorporation could influence bilayer properties such as fluidity, permeability, size, and surface charge, potentially affecting encapsulation and retention. The phenolic profile differences between ‘Bidni’ and ‘Bajda’ further support the notion that liposomes derived from different cultivar extracts can vary in structural integrity, impacting membrane stability and retention of encapsulated compounds. These findings underscore the role of cultivar-specific matrix compositions in determining encapsulation efficiency and liposome performance.

Flavonoids such as quercetin (XLogP = 1.5) and tetrahydroxyflavone (XLogP = 2.5), abundant in the extracts, also exhibit high lipophilicity, making them more efficiently encapsulated by Method A. This is reflected in the elevated total flavonoid content (TFC) encapsulation efficiency. Furthermore, apigenin 7-glucoside, despite its low lipophilicity (XLogP ≈ 0), displays amphiphilicity resembling that of phosphatidylcholine, with hydrophilic heads and a lipophilic center. This structural similarity likely facilitates its integration into lipid bilayers, further supporting Method A’s superior performance for encapsulating flavonoids.

Statistically significant differences in encapsulation efficiencies (EE%) were observed between the ‘Bidni’ and ‘Bajda’ cultivars. Most notably, ‘Bajda’ exhibited a 15% higher CUPRAC EE% and a 6% increase in TdOPC EE% compared to ‘Bidni’. Additionally, ‘Bajda’ showed a higher mean TPC EE% by 6%, although this difference was not statistically significant. In contrast, flavonoid encapsulation efficiencies were similar across both cultivars, with no significant differences. These findings suggest that the matrix composition of each cultivar extract influences the encapsulation process, likely by altering the liposomal characteristics. In fact, another study [32] has previously highlighted that high phenolic concentrations can reduce EE%.

Figure 5 illustrates the superior cytotoxicity observed in liposomal extracts encapsulated using Method A, reflected by a significantly lower IC_50_ value compared to Method B (*p* < 0.05); this can be attributed to the higher encapsulation efficiency of flavonoids. Flavonoids, such as quercetin and tetrahydroxyflavone, are well-known for their cytotoxic effects, including apoptosis induction, enzyme inhibition, and disruption of cancer cell signaling pathways. The mechanism of Method A, which embeds lipophilic flavonoids directly into the lipid bilayer, aligns with their lipophilic nature, facilitating stabilization and retention within the bilayer. While hydrophilic phenolics encapsulated more effectively in Method B may have lower cytotoxic potential, the flavonoid enrichment in Method A likely contributed significantly to the cytotoxic outcomes. Additionally, the structural properties of liposomes formed by Method A may enhance cellular uptake or release kinetics of these bioactive compounds, amplifying their effects. The comparison between encapsulated and non-encapsulated extracts revealed notable findings. At 24 h of incubation, the non-encapsulated extract exhibited a significantly lower IC_50_ value compared to encapsulated extracts (*p* < 0.05), with pairwise comparisons confirming a significant difference between the non-encapsulated extract and Formulation 1 (*p* < 0.05). However, after 48 h, the encapsulated extracts demonstrated superior cytotoxicity, with both Formulation 1 and Formulation 2 showing lower IC_50_ values than the free extract (*p* < 0.05). Collectively, these findings suggest that while non-encapsulated extracts exert an acute cytotoxic effect, encapsulated extracts are more effective over prolonged exposure. This time-dependent shift aligns with the literature findings [33,34,35,36,37], which report enhanced cytotoxicity of bioactive compounds when delivered via liposomal encapsulation. Another author [38] further corroborates this phenomenon, demonstrating that non-purified liposomal formulations initially exhibit greater cytotoxicity due to free drug content but become more effective over time as purified liposomal formulations dominate. The delayed cytotoxicity of encapsulated extracts may be attributed to the time required for liposomes to adhere to and fuse with cancer cell membranes, releasing bioactive substances intracellularly. This process contrasts with the free extract, which diffuses directly across the cell membrane but is constrained by solubility, concentration gradients, and polar/non-polar interactions. Liposomes also employ a secondary mechanism, endocytosis, wherein gradual diffusion from intracellular vesicles contributes to sustained cytotoxic effects.

## 4. Materials and Methods

### 4.1. Preparation of Olive Mill Waste Extracts

A OMW composite (pomace and water) derived from two distinct Maltese olive cultivars, namely ‘Bidni’ and ‘Bajda’, was obtained from local olive oil producers, which were equipped with both 2-phase and 3-phase centrifugation systems. The composite was also composed of samples collected at different stages of olive maturity to further diversify phenolic compound composition. The methodology used for methanolic extract preparation of both cultivars is identical to what was used in another study [38].

### 4.2. Liposomal Encapsulation of OMW Extracts

Liposomal encapsulation of the ‘Bidni’ and ‘Bajda’ cultivars was performed using the Bangham thin-film hydration method [39], which typically yields multilamellar vesicles (MLVs). Although MLVs can efficiently encapsulate both hydrophilic and lipophilic compounds, they often produce polydisperse size distributions. To reduce lamellarity and vesicle size, we extruded the suspensions 35 times at 55 °C through a 0.4 µm polycarbonate membrane (Avanti Polar Lipids, Alabaster, AL, USA). Because membranes with pore sizes larger than 0.2 µm generally do not yield unilamellar liposomes, the final formulations likely remain multilamellar and somewhat polydisperse. Despite this, the approach provides straightforward preparation, high encapsulation efficiency, and reliable delivery for complex phenolic extracts. In brief, soybean lecithin (≥94% phosphatidylcholine, <2% triglycerides; VWR, UK) and cholesterol (Biochem Chemopharma, France) were dissolved in chloroform. Two phosphatidylcholine-to-cholesterol ratios were examined:

Formulation 1 (80:20), to investigate the effect of reduced cholesterol;

Formulation 2 (70:30), a conventional 2:1 ratio noted in the literature [39].

Following rotary evaporation of the solvent, the resulting thin lipid film was freeze-dried. Separately, we prepared the phenolic extract by dissolving 10 g of freeze-dried OMW powder in 100 mL of methanol, generating a 100 mg/mL stock solution. An appropriate volume of this concentrate was then diluted in PBS to obtain 10 mg/mL, the target concentration for liposomal hydration.

To form the liposomes, we used two methods:

Method A: The phenolic extract was added directly to the lipid–chloroform mixture before solvent removal, promoting bilayer formation around the extracts.

Method B: The phenolic extract in PBS was added to the dried lipid film, allowing passive diffusion of extracts into empty vesicles. Table 3 summarizes the design of experiments carried out in this study.

After extrusion, the liposomal suspensions were sterilized by passage through a 0.22 µm PES syringe filter in a laminar flow hood and stored at 4 °C in sterile microcentrifuge tubes for short-term use.

### 4.3. Determination of Encapsulation Efficiency

To evaluate the encapsulation efficiency with respect to different phenolic compounds and other reducing agents, a comparison was conducted between the total content present in the analyzed volume, serving as the 100% reference point, and the content that remains unbound and freely dispersed in the solution. This comparison enabled the assessment of the encapsulation efficiency achieved as a % of the total. To achieve an accurate initial reference point, the liposomal solution itself was utilized instead of diluting the methanolic stock to 10 mg/mL. This methodology was chosen to account for any minimal losses that might have taken place during the liposomal encapsulation step. Furthermore, it aimed to ensure that both the reference point and the resulting unbound matrix were generated from the identical sample and under the same conditions. The reference point was generated by following a methodology used in another study [40], which involved de-emulsification of the lipids with the use of isopropyl alcohol, acidified with hydrochloric acid. In contrast to other known methodologies, separation of the free extract was achieved using the centrifree method as described in a previous study [41]. The procedure involved subjecting the liposomal sample to centrifugal filtration at high rpm, which resulted in the free compounds along with the medium passing through the membrane and into the bottom of the centrifuge tube. However, due to size exclusion, the liposomes are retained in the filtration membrane, hence achieving a purified sample which is free of lipid vesicles. Furthermore, the same authors [41] proposed sample dilution to achieve accurate results, and hence a lipid dilution of 10-fold was adopted for the purpose of this study to achieve accurate results. To ensure uniformity, this dilution was therefore also applied to the de-emulsified reference samples. Prior to all chemical assay quantifications, all raw absorbance values underwent the subtraction of their respective blank absorbance values. The centrifree blank was prepared using complete PBS, whereas the de-emulsified blank was prepared by mixing PBS and acidified isopropanol in a ratio of 1:10 *v*/*v*. Encapsulation efficiencies were determined using the equation:

(1) $$EE\%=\frac{{DEM\;Sample}_{ABS}\;-\;{Centrifree\;Sample}_{ABS}}{{DEM\;Sample}_{ABS}}\times 100$$

### 4.4. Determination of Total Phenolic Content

TPC determination for all liposomal samples was conducted using the Folin–Ciocalteu colorimetric method. Aliquots (20 μL) of de-emulsified, centrifree, and 10-fold diluted samples were transferred to a 96-well plate, oxidized with 100 μL of 5-fold diluted FC reagent, and alkalized with 80 μL of 7.5% Na_2_CO_3_ (pH 10). After 1 h in the dark, the plate was agitated and absorbance measured at 630 nm using a microtiter plate reader. A calibration curve was prepared using gallic acid in methanol at concentrations of 200–5 mg/L. After correcting for methanol absorbance, a linear regression model (R^2^ = 0.9935 y = 0.0043x + 0.0138) determined the LOQ (11.99 mg/L) and LOD (3.95 mg/L)

### 4.5. Determination of Total Flavonoid Content

The determination of TFC for all liposomal samples was achieved using a method employed in another study [42], with slight in-house modifications. Aliquots (25 μL) of 10-fold diluted, de-emulsified, and centrifree samples were added to a 96-well plate, followed by 10 μL each of 10% aluminum chloride and 7% sodium nitrite, and 80 μL of deionized water. After 30 min, 100 μL of 1M NaOH was added, and the plate was agitated before measuring absorbance at 450 nm. Absorbance values were converted to quercetin equivalents (mg/L) using an 8-point calibration curve (5–200 mg/L), yielding a linear regression (R^2^ = 0.9742, y = 0.0008x − 0.0006). LOQ and LOD were determined as 26.09 mg/L and 8.61 mg/L, respectively.

### 4.6. Determination of Total Ortho-Diphenolic Content

The determination of TdOPC for liposomal samples was based on a modified version of Arnow’s colorimetric method [43]. Arnow’s reagent was prepared by dissolving 25 g each of sodium nitrite and sodium molybdate dihydrate in 250 mL ethanol/water (1:1 *v*/*v*). Aliquots (20 μL) of 10-fold diluted, de-emulsified, and centrifree samples were added to a 96-well plate, followed by 20 μL of 1M HCl and brief mixing. Next, 20 μL of Arnow’s reagent was added, and the plate was agitated vigorously. Subsequently, 80 μL of deionized water and 40 μL of 1M NaOH were added. After a final agitation, absorbance was measured at 405 nm. Results were expressed as protocatechuic equivalents (mg/L) using an 8-point calibration curve (5–200 mg/L). The curve, constructed with protocatechuic acid in methanol, yielded a linear regression (R^2^ = 0.9595 y = 0.0008x + 0.007). LOQ and LOD were calculated as 33.64 mg/L and 11.09 mg/L, respectively.

### 4.7. Determination of CUPRAC

The determination of CUPRAC for liposomal samples followed a modified version of the standard method [44]. Aliquots (20 μL) of 10-fold diluted, de-emulsified, and centrifree samples were added to a 96-well plate, followed by 100 μL of 10 mM copper(II) chloride, 100 μL of 1M ammonium acetate (pH 7.0), and 100 μL of 7.5 mM neocuproine in ethanol. The plate was agitated briefly, left to stand for 30 min, and absorbance was measured at 405 nm. Results were expressed as gallic acid equivalents (mg/L) using an 8-point calibration curve (5–200 mg/L). The calibration curve, prepared with gallic acid in methanol, provided a linear regression (R^2^ = 0.9964R y = 0.0076x + 0.0037). LOQ and LOD were calculated as 9.88 mg/L and 3.26 mg/L, respectively.

### 4.8. Cell Culture Seeding

An HL-60 cryovial stored at −80 °C was rapidly thawed in a lukewarm water bath after disinfection with 70% ethanol. The thawed cells were transferred dropwise into cold medium, centrifuged at 1200 rpm for 5 min, and the DMSO layer was discarded. The pellet was resuspended in 1 mL of fresh medium, transferred to a T_25_ flask with 37 °C medium, and incubated at 37.5 °C with 5% CO_2_, with flask caps loosened for gaseous exchange. Single-cell suspensions were prepared by pipetting to disperse clumps, and cell counting was performed using a hemocytometer. The suspension was diluted to 8000 cells per well in 180 µL of medium, and cells were seeded into 96-well plates with 180 µL of 10% FBS medium added to each well.

### 4.9. Cytotoxicity Testing

At 4 h prior to the designated 24- and 48-h exposure points, 20 µL of a 5 mg/mL MTT solution was added to each well. The MTT solution was prepared by dissolving 1 g of MTT powder in 200 mL of phosphate-buffered saline (PBS), followed by filter sterilization and overnight storage. After MTT addition, plates were incubated for 4 h and inspected for formazan crystal formation, then centrifuged at 2400 rpm for 5 min. The supernatant was removed, and 100 µL of DMSO plus 25 µL of glycine buffer (pH 10.5) were added to each well.

Absorbance was measured at 650 nm using a microtiter plate reader, and dose–response curves were subsequently constructed by plotting the natural logarithm of extract concentration (x-axis) against cell viability (% of control) (y-axis). A logarithmic best-fit curve was applied in the form:

(2) y=aln(x)+b

where x is the extract concentration, y is the cell viability, and ln denotes the natural logarithm (base e). The parameters a and b were determined via least-squares regression, minimizing the sum of squared deviations between observed data points and the fitted curve. The resultant model was used to identify the IC_50_, defined as the extract concentration at which cell viability declined to 50% of the untreated control. This approach linearizes the dose–response relationship, facilitating a straightforward determination of the half-maximal inhibitory concentration.

## 5. Conclusions

This research highlights the multifaceted potential of liposomal encapsulation as an advanced drug delivery system, highlighting its ability to enhance the encapsulation efficiency, stability, and cytotoxicity of bioactive compounds. The findings demonstrated that encapsulation mechanisms are significantly influenced by the physicochemical properties of phenolic compounds, including their hydrophilicity, lipophilicity, and structural amphiphilicity, as well as the methods and formulations used. Method A proved more effective for encapsulating lipophilic flavonoids, aligning with its superior cytotoxicity outcomes, while Method B was better suited for hydrophilic phenolics, achieving higher encapsulation efficiency for total phenolic compounds. Additionally, cultivar-specific differences, particularly between ‘Bidni’ and ‘Bajda’, revealed that matrix composition can significantly impact encapsulation efficiency and liposomal properties. The time-dependent cytotoxic effects of encapsulated extracts further highlighted the advantages of liposomal delivery, with encapsulated formulations outperforming free extracts over prolonged exposure due to their controlled release mechanisms and enhanced cellular uptake through membrane fusion and endocytosis.

These findings align with and expand upon the existing literature, emphasizing the importance of optimizing liposomal formulations and encapsulation strategies to achieve tailored therapeutic outcomes. The demonstrated ability of liposomes to provide sustained release and targeted delivery of bioactive compounds highlights their potential as a cornerstone in developing next-generation drug delivery systems for enhanced efficacy and reduced side effects. Future research should focus on refining liposomal formulations for specific compound profiles and exploring their clinical applications in targeted therapies.

## Figures and Tables

**Table 1:** Quantification of Selected Bioactive Compounds in Methanolic Extracts of Bajda and Bidni Olive Varieties [5].

Compound	Peak number	Bajda Methanol (μg/mL)	Bidni Methanol (μg/mL)
5,7-dihydroxy flavone	15	<LOD	1.641 ± 0.030
Quercetin	14	2.064 ± 0.001	<LOD
3′,4,5,7-tetrahydroxy flavone	13	3.551 ± 0.001	<LOD
Ligstroside	12	1.303 ± 0.013	0.286 ± 0.006
Oleocanthal	11	1.284 ± 0.069	<LOD
Oleuropein	10	6.265 ± 0.042	2.398 ± 0.240
Oleacein	9	11.085 ± 0.021	5.651 ± 0.126
Apigenin 7-glucoside	8	8.109 ± 0.178	3.343 ± 0.129
Ellagic acid	7	0.218 ± 0.001	<LOD
p-coumaric acid	6	2.348 ± 0.013	1.927 ± 0.007
Vanillin	5	4.084 ± 0.001	<LOD
Vanillic acid	4	2.011 ± 0.013	<LOD
2-(4-hydroxy phenyl) ethanol	3	3.763 ± 0.010	3.608 ± 0.019
3-Hydroxytyrosol	2	6.034 ± 0.044	6.801 ± 0.312
Gallic acid	1	0.505 ± 0.006	<LOD

**Table 2:** Physicochemical Properties of Selected Bioactive Compounds.

Compound Name	TPSA (Å²)	XLogP	Hydrophilicity/Lipophilicity
Tetrahydroxyflavone	107	2.5	Moderately lipophilic
Quercetin	127	1.5	Mildly lipophilic
p-coumaric acid	57.5	1.5	Balanced hydrophilic-lipophilic profile
Vanillin	46.5	1.2	Slightly lipophilic
Oleacein	101	1.1	Mildly lipophilic
2-(4-hydroxyphenyl) ethanol	40.5	0.4	Slightly hydrophilic
Apigenin 7-glucoside	166	−0.1	Hydrophilic
Oleuropein	202	−0.4	Strongly hydrophilic
3-hydroxytyrosol	60.7	−0.7	Highly hydrophilic

**Table 3:** Experimental matrix showing the combination of olive oil cultivars (‘Bidni’ and ‘Bajda’), formulation ratios (80:20 and 70:30), and processing methods (Method A and Method B) used for each treatment group. Each cultivar was subjected to both formulations and both methods, resulting in eight distinct treatment conditions (2 × 2 × 2) for three independent replicates.

	‘*Bidni*’ Cultivar	‘*Bajda*’ Cultivar
Formulation 1 (80:20)	Method A	Method A
	Method B	Method B
Formulation 2 (70:30)	Method A	Method A
	Method B	Method B

## Data Availability

Data is contained within the article and the Supplementary Materials.

## Supplementary Materials

The following supporting information can be downloaded at: https://www.mdpi.com/article/10.3390/molecules30112351/s1; Figure S1: Calibration curve used for the determination of total phenolic content; Figure S2: Calibration curve used for the determination of total flavonoid content; Figure S3: Calibration curve used for the determination of total ortho-diphenolic content; Figure S4: Calibration curve used for the determination of CUPRAC.

## Abbreviations

The following abbreviations are used in this manuscript:

TPC	Total Phenolic Content
TFC	Total Flavonoid Content
TdOPC	Total Ortho-Diphenolic Content
CUPRAC	Cupric Reducing Antioxidant Capacity
PC	Phosphatidylcholine
Chol	Cholesterol
PBS	Phosphate-Buffered Saline
EE%	Encapsulation Efficiency Percentage
OMW	Olive Mill Waste
OMWW	Olive Mill Waste Water
MTT	3-(4,5-dimethylthiazol-2-yl)-2,5-diphenyltetrazolium bromide (used in cytotoxicity assays)
IC_50_	Half Maximal Inhibitory Concentration
DMSO	Dimethyl Sulfoxide
HL-60	Human Leukemia Cell Line
LOQ	Limit of Quantification
LOD	Limit of Detection
TPSA	Topological Polar Surface Area
XLogP	Partition Coefficient (Lipophilicity Measure)
