# Supplementary material for: Formulation and Evaluation of Liposome-Encapsulated Phenolic Compounds from Olive Mill Waste: Insights into Encapsulation Efficiency, Antioxidant, and Cytotoxic Activities
Source: Molecules. 2025 May 28;30(11):2351. doi: 10.3390/molecules30112351 (PMC12156456; doi:10.3390/molecules30112351)
Supplement: Supplementary file 1 [file molecules-30-02351-s001.zip › molecules-3512958-supplementary.pdf]

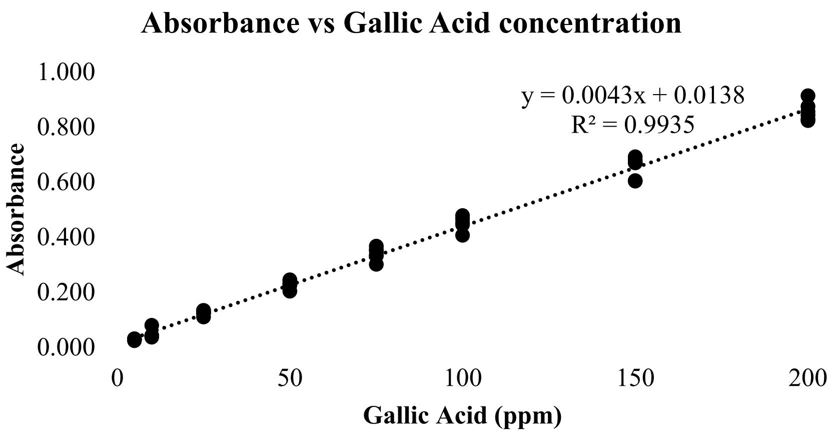

Figure S1: Calibration curve used for the determination of total phenolic content

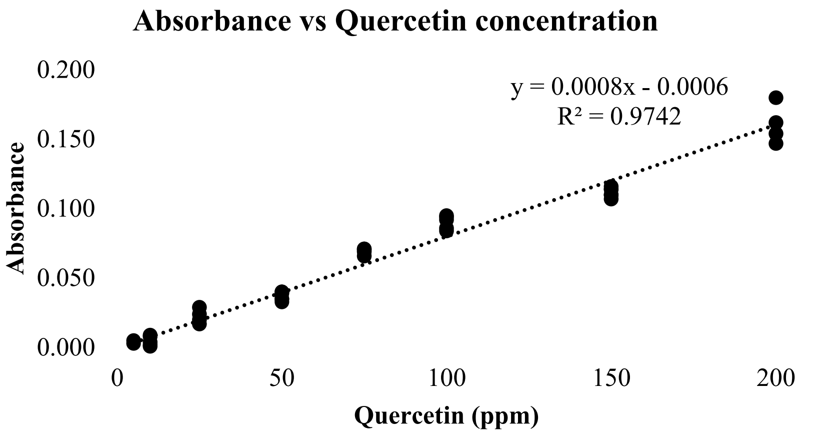

Figure S2: Calibration curve used for the determination of total flavonoid content

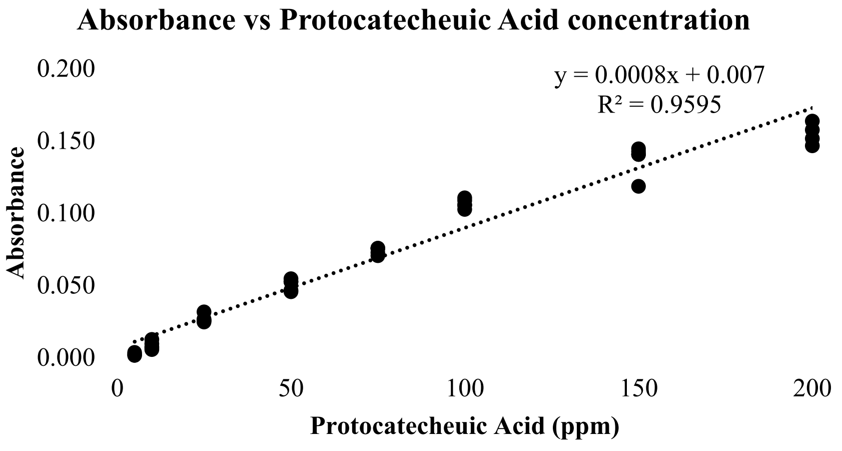

Figure S3 Calibration curve used for the determination of total orthodiphenolic content

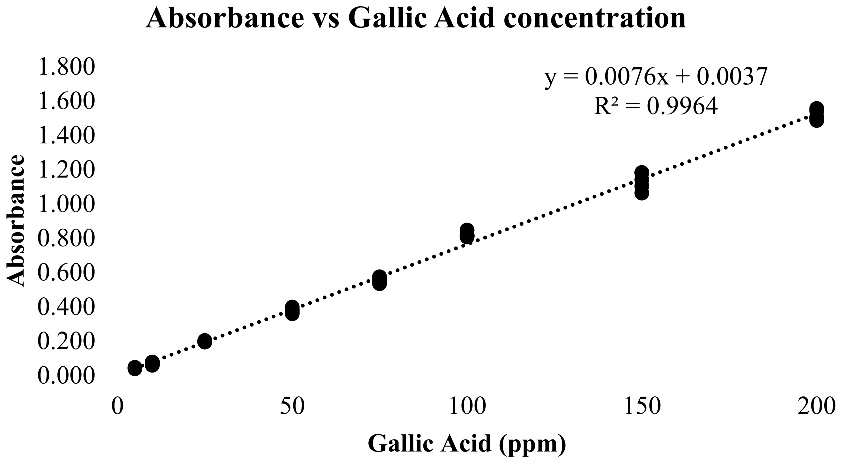

Figure S4: Calibration curve used for the determination of CUPRAC.
